# Family Resilience in the Oncology Setting: Development of an Integrative Framework

**DOI:** 10.3389/fpsyg.2018.00666

**Published:** 2018-05-08

**Authors:** Flavia Faccio, Chiara Renzi, Alice V. Giudice, Gabriella Pravettoni

**Affiliations:** ^1^Department of Oncology and Hemato-Oncology, University of Milan, Milan, Italy; ^2^Applied Research Division for Cognitive and Psychological Science, European Institute of Oncology, Milan, Italy

**Keywords:** family resilience, cancer, psychological, oncology, model, framework

## Abstract

Resilience is a concept that has received burgeoning interest in the last decades. Researchers have been fascinated by the ability of some individuals to bounce back from an adverse event and adapt to extremely challenging situations. More recently family resilience, namely the potential resources of the family’s system, has been considered due to numerous individual studies highlighting the crucial influence of relationships with significant others in mediating adaptation and recovery. In this article, a brief overview of the theoretical literature on individual and family resilience is presented. Following, current empirical literature on resilience in the context of oncology is evaluated. Although family resilience is considered a dynamic process unique to each family unit, some common resources and strengths that can help families face significant adversities, such as cancer, can be identified. This said to date there is no family resilience framework applied specifically to the cancer trajectory. Drawing from previous studies, we sought to provide a clinical resilience model for families living with cancer, with the aim of mapping those resources that can empower families to deal with prolonged adversity. This framework can serve as general guideline for health professionals in assessing family strengths, promoting specific family processes and facilitating adaptation to the cancer experience.

## Resilience: A Multifaceted Process

Resilience is a complex concept with no universally accepted definition, however, important commonalities have been identified (Aburn et al., 2016). One of the most classical definitions describes resilience as the ability to bounce back and adapt successfully despite challenging situations (Rutter, 2006). A recent concept analysis of resilience suggests that there are three requirements for resilience to happen: a situation of significant adversity, the presence of a number of resources that can face the adversity and facilitate adaptation and the avoidance of a negative outcome or a successful adaptation to the new situation (Windle, 2011). Masten (2001) describes resilience as “ordinary magic,” to emphasize that it is inherent in all of us and that it is difficult to measure or quantify. The experience of resilience varies across the individual’s lifespan and the presence of a potentially stressful event, such as cancer, can modify one’s ability to be resilient (Windle, 2011).

Within the cancer continuum, resilience has been described as a baseline characteristic, an outcome and as a mechanism that can promote positive growth (Molina et al., 2014). Several studies have shown that resilience is higher in cancer patients than the general population (Wenzel et al., 2002; Strauss et al., 2007; Min et al., 2013; Rosenberg et al., 2014) and that it is independent from demographic and illness-related variables (Strauss et al., 2007). Resilience correlates with lower emotional distress, even when controlling for possible confounding factors (Min et al., 2013). According to Strauss et al. (2007) higher resilience may be due to higher awareness and activation of personal resources to face the illness. Wenzel et al. (2002) also suggest that cancer survivors’ use of coping strategies and confidence in managing survivorship issues were predictors of psychological adjustment.

## A Systemic Perspective: Shifting From the Individual to the Family

A cancer diagnosis affects not only the single individual but also the family system, which can support positive adaptation through the activation of resilience processes or succumb to the weight of the illness. The family systems theory considers individuals as part of a unit woven with emotions, where they are inseparable from the system (Broderick, 1993). Therefore, changes in one member of the family would provoke alterations to the whole system, which may move smoothly to a new homeostasis incorporating the new information, namely the cancer diagnosis, or encounter difficulties in the readjustment process. The systemic approach has progressively abandoned the view of troubled families as “damaged” and moved onto a positive view of the family as challenged by adversities but able to overcome them (Walsh, 2003). There has also been a shift from viewing the family as a relational context in which individual resilience manifests itself to considering family resilience as a unit with family-level constructs that influence individuals (Becvar, 2012). Within this framework it is possible to view cancer as a “family disease” as it affects not only the person diagnosed with cancer and their loved ones individually, but also their relationships and the family functioning (Rolland, 2005).

## Family Resilience – Definitions and Models

Three groups of authors have contributed substantially to the definition and implementation of this field of research (McCubbin and McCubbin, 1988; Hawley and DeHaan, 1996; Walsh, 2003). McCubbin and McCubbin (1988) describe it as a set of dimensions and characteristics that help the family become “resistant to disruption in face of change and adaptive in the face of crisis situations” (p. 247). Hawley and DeHaan (1996) define family resilience as a path during which the family “adapts and prospers in the face of stress, both in the present and over time” and responds in a unique way depending on a combination of internal/external protective and risk factors. While McCubbin and McCubbin (1988) describe resilience as a family characteristic, Hawley and DeHaan (1996) stress the existence of developmental phases. Finally, Walsh (2016, 2003) defines it as the ability to “withstand and rebound from adversity, strengthened and more resourceful,” thus encompassing personal growth.

Research in this area has developed around two major models: Resiliency Model of Family Adjustment and Adaptation (RMFAA)(McCubbin and McCubbin, 1993) and the Family Resilience Framework (Walsh, 2003). McCubbin and McCubbin (1993) divide resilience processes in two phases. During the Adjustment phase, families strive to achieve a balance after the stressful event. This depends on a number of factors, including the type of stressor, its severity, vulnerability to a particular stressor and the family’s established patterns of functioning. During the Adaptation phase instead the family conducts a broad and specific appraisal process of themselves and of the stressor (Becvar, 2012). According to the authors, families need to progress through specific steps in order to achieve resilience. Developed with a specific adversity, in mind, this model is quite rigid in face of the wide range of stressful events observed nowadays. Nevertheless, it offers insight into the phases of adjustment and adaptation that are key to understanding resilience processes in cancer.

Coming from a family system approach, Walsh (2003, 2016) presents a more flexible framework for family resilience, removing specific categories and recognizing the uniqueness of each family unit. Functioning is assessed within each family context, considering their values and resources within three major dimensions: (i) belief systems, (ii) organizational patterns and (iii) communication processes. These are considered mutually interactive and synergistic as they facilitate and sustain each other across systems and over time. One of the advantages of Walsh’s model is the utility of this framework for clinical prevention and intervention, applied to contexts ranging from those recovering from state of terror (Griffith et al., 2005) to those living with childhood illness or disability (Rolland and Walsh, 2006). This said the model does not investigate in depth the possibility of different resilience processes according to the phase of illness.

## Families Living With Cancer

To date, research addressing family resilience in oncology is scant (Van Schoors et al., 2015). Most of the research focuses on families with an ill child rather than an ill parent. For example, Lee et al. (2004) assessed coping strategies, adaptation and resources of parents with an ill child, noticing that families who thrived in spite of the child’s illness mentioned open communication, mutual understanding, flexibility in reorganizing the family, attachment, and balance as recurrent themes. Another qualitative study instead used the RMFAA to identify resilient family properties (McCubbin et al., 2002). Parents highlighted rapid mobilization and reorganization of childcare, appraisal and reflection on the cancer experience, followed by support from the healthcare team and from the extended family. Working together, dividing roles, being emotionally available and adapting to changes in family functioning were vital family strengths to cope with childhood cancer. In addition to this, giving meaning to their child’s cancer and to the changes occurring in their lives allowed parents to become aware of the possibility of fostering family cohesiveness in face of an adverse event (McCubbin et al., 2002). Another study conducted with fathers revealed that adjustment in family life and communication patterns were predictors of resilience processes (Brody and Simmons, 2007). Finally, it is worth mentioning another qualitative study in which fathers considered resilience as the need to adapt to change through stabilization of family life, and incorporate new family routines and rituals into cancer care (Buchbinder et al., 2009).

From the findings presented above, it seems that the activation of resilience processes in face of cancer diagnosis and treatment allow to overcome daily stressors and to reach a new balance in family functioning. However, when a family struggles with the challenges posed by cancer there is a risk of functioning in a chaotic manner and of diverting its life course (Kazantzaki et al., 2018). This translates into clinically significant levels of distress, higher risk of developing psychosocial problems and low family cohesion (Weihs and Reiss, 2000).

Therefore, from an oncological perspective, the quality of the family’s relational functioning can affect the family members’ adjustment to the life-threatening illness (Kazantzaki et al., 2016) and modulate outcome and survival (Kroenke et al., 2006; Nausheen et al., 2009). As close relationships can influence the cancer trajectory, it is vital to address and strengthen relational aspects that can positively influence adjustment and outcome of the illness.

## Applying a Family Resilience Framework to the Cancer Trajectory

While it seems vital to offer a clinical framework to promote resilience processes in families living with cancer, it has been applied very little in oncological settings. The only attempt at offering an integration between family resilience and somatic disease is the application of a family systems resilience framework in childhood illness and disability (Rolland and Walsh, 2006).

**FIGURE 1:**
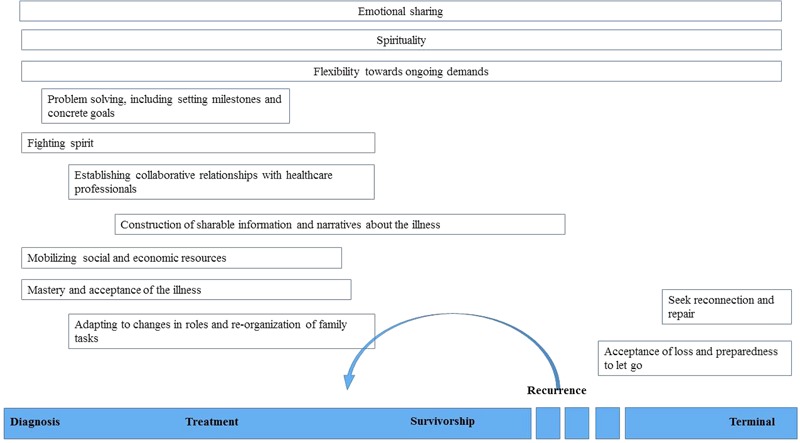
Family resilience in the cancer trajectory.

Starting from Walsh’s (2003) framework we propose a model of family resilience in cancer care which encompasses twelve key processes. Within this framework it is imperative to consider the trajectory of cancer, which is composed of different, and possibly recursive, phases: diagnosis, treatment, survivorship, recurrence, and terminal illness (Molina et al., 2014). Although no study has assessed specific family resilience processes according to the illness phase it is essential to critically evaluate which processes could be considered specific to some phases and which may be common to the whole trajectory. Following previous studies and clinical experience in the oncology setting it seems that emotional sharing, flexibility to ongoing demands and spirituality are resilience processes that can be activated during any step of the cancer continuum. The other key resources instead are specific to one or more time phase and are presented in **Figure 1**.

When a cancer diagnosis is communicated to the family, an acute crisis phase begins. During the first couple of weeks, the lack of clinical information about tumor stage and prognosis throws the family in a state of shock and paralysis where fear of losing the family member prevails. Perceived loss of control over their lives is heightened by feelings of confusion and anguish. Fostering a positive outlook while keeping in mind the reality of the disease may increase the family’s ability to recover and cope (Walsh, 2016). The clinician can support the family in this delicate moment by focusing on the here-and-now and, at the same time, delineate a plan of what should be expected in terms of treatment plan, prognosis and course of cancer (Rolland, 2005). It is possible to create a comfortable space for the family to ask questions in order to contain their feelings and recast the health challenge as comprehensible. Normalizing and contextualizing these challenges allows the family to perceive their strains and emotional states as understandable and common to other families, which ultimately increases their sense of competence. Spiritual resources, deep faith and rituals can bring strength and comfort to the family, ultimately nourishing resilience (Walsh, 2003).

During the treatment phase flexibility toward ongoing demands, open communication, collaborative relationship are some of the family resilience processes that facilitate adaptation to cancer care. As the treatment phase is characterized by uncertainty, roles might continue to shift as the disease unfolds, thus requiring openness to change and adaptation. Both flexibility to change and sense of continuity are key aspects that bring strength and comfort to the family. The former guarantees adaptation to different roles, for instance a caregiver can become someone to take care of. The latter is provided by routine maintenance, which helps the family members to regain a sense of normalcy. Temporary separation from the ill member can be experienced as a loss due to decreased emotional availability (Buchbinder et al., 2009). Family routines are disrupted, commitments are re-prioritized and new caregiving arrangements are put in place in order to accommodate illness management (Walsh, 2013).

In this phase clinicians should try to provide a bridge between the biological world of the illness and the psychosocial one of the family (Rolland, 2005). A patient might need time to process illness management; however, if there is no time, a family member can act as a temporary proxy who holds the necessary medical information in order to carry out medical routines at home. Clear and consistent communication between members and possibility to share feelings and fears can help the family to construct a shared story that organizes their experience and allows them to gain a sense of coherence (Rolland and Walsh, 2006). If there are barriers to shared communication, clinicians can support the family by helping them develop their own implicit meaning of the cancer-event and integrate it in the family narrative. During this phase transitions in the family life cycle, such as an adolescent’s strive for independence and permission to move in and out of the family system, should not be put on hold for too long, rather they should be encouraged to avoid feelings of guilt and blame (Rolland, 2005).

Survivorship is a phase during which cancer patients could be tackling long-term medical and psychosocial consequences of the disease with an indirect impact on adaptation to life after cancer. Some families might instead return smoothly on a cancer-free family course, which differs from their life course prior to diagnosis, and may manifest personal and relational growth. Organizational patterns may change as re-appraisal of family priorities and values occurs.

Recurrence can be experienced as a second, acute crisis phase, which requires reorganization and re-adaptation. Patients and their families might experience loss of hope for recovery and fear of death of the family member. This said, levels of confusion and anxiety are lower than those experienced at initial diagnosis as there is less uncertainty and more knowledge about the cancer treatment-related challenges (Yang et al., 2008). Clinicians could inquire about those strategies that have been activated in the past and reconsider their areas of vulnerability to predict the family’s fit to the health challenges ahead of them. Past support networks can be activated more readily and help the patient maintain meaning in life. As cancer therapies in this phase might be more aggressive and therefore influence severity of physical symptoms, physicians should communicate clearly the possible constraints and functional limitations that the patient and his/her family might encounter. Acceptance of what is beyond control and what cannot be changed allows the family to reach collaborative decisions with the medical team about the best treatment options.

During the terminal phase instead, family members are forced to cope with definitive separation and mourning. It is important to plan disclosure of the terminal stage to the patient and their family, as inappropriate disclosures by physicians or learning bad news indirectly are known to increase distress (Yun et al., 2010). Psychologists can investigate the emotional responses of patients and of caregivers to the disclosure of the terminal stage and evaluate whether they pertain to an expected grief process. Families cope best when they shift from the previous perspective of being able to control the disease to letting go of this sense of control, thus easing their suffering (Rolland and Walsh, 2006). For the patient instead, the prospect of no more suffering and dying peacefully may help them cope. Moreover, the feeling that they are not a burden anymore to their families allows for adjustment to this phase (Yun et al., 2010). At this time it is essential to make the most out of the time spent together, to try and reconnect and repair relationships with significant others (Rolland, 2005). They also have to make important decisions about when to introduce palliative and/or hospice care. Healthcare professionals can help with practical tasks, evaluate the family’s awareness of the impending loss and aid them in reorganizing their family unit (Rolland and Walsh, 2006). Extended kin can be a vital social resource as they can provide support in dealing with childcare and other practical chores. In this last phase, setting attainable and appropriate goals together, such as improved quality of life rather than fostering hope for a cure can be perceived as an opportunity to achieve last wishes and live as fully as possible.

## Future Directions

Starting from these premises, some future directions for research and clinical practice can be considered. First, development of assessment measures that evaluate needs and expectations of family members would promote a family-centered model of care. Besides administering these to all family members, assessment of the level of agreement between members regarding their psychosocial needs should be undertaken. This would allow for targeted interventions on specific disease-related aspects and distresses that might be overlooked by other members of the family. As the doctor often has a patient-centered view of the illness, which does not take into account relatives or caregivers, elements of system theory should be introduced in medical training in order to increase awareness about the influence of the family on the patient’s decisions and overall wellbeing. Finally, an increasing number of studies has investigated the cancer patient’s emotional state and general psychological functioning, while only recently attention has been given to the caregiver’s and family member’s emotional and physical wellbeing (Swore Fletcher et al., 2008; Stenberg et al., 2009; Bevans and Sternberg, 2012). These studies have shown that the emotional burden carried by caregivers of cancer patients can increase their levels of depression, fatigue and poor sleep quality (Stenberg et al., 2009), which in turn decrease immunocompetence (Bennett et al., 2013). Further research into the caregiver’s and family’s wellbeing when caring for a close relative with cancer will help healthcare professionals develop interventions and support the family during the cancer trajectory, preventing onset of psychological difficulties and of physical symptoms, which might develop into psychosomatic illnesses.

## Conclusion

Keeping in mind that resilience is a unique, developmental trajectory for each family and may differ according to the type of cancer, this model aims to provide a map of the main resources that allow families to overcome and potentially grow from the experience of cancer. Moreover, it can give useful indications to health care professionals on how to support the development of resilience in moments of crisis. Further studies should investigate which relational resilience processes can help the family deal with cancer and develop evidence-based interventions for families that struggle and are at risk of functioning in a maladaptive way.

## Author Contributions

FF and CR made substantial contributions to manuscript conception. FF and CR drafted the manuscript while AG and GP revised it critically providing vital intellectual contributions. All authors approved its final version.

## Conflict of Interest Statement

The authors declare that the research was conducted in the absence of any commercial or financial relationships that could be construed as a potential conflict of interest.
